# The Use of Griffiths III in the Appraisal of the Developmental Profile in Autism: A Systematic Search and Review

**DOI:** 10.3390/brainsci15050506

**Published:** 2025-05-15

**Authors:** Flavia Lecciso, Chiara Martis, Annalisa Levante

**Affiliations:** 1 Department in Human and Social Sciences, University of Salento, 73100 Lecce, Italy; flavia.lecciso@unisalento.it (F.L.); chiara.martis@unisalento.it (C.M.); 2 Lab of Applied Psychology, Department in Human and Social Sciences, University of Salento, 73100 Lecce, Italy

**Keywords:** Griffiths III, autism spectrum disorders, developmental profile, autism intervention, STROBE, JBI, CEC

## Abstract

Background: Griffiths III is a child-friendly and play-oriented direct gold-standard measure of a 0–6-year-old child’s developmental profile. It is a measure that helps practitioners in detecting the weaknesses in children who have an increased likelihood or a diagnosis of neurodevelopmental disorders, e.g., autism. Objectives: Following the PICO protocol, two research questions addressed the current systematic search and review (Prospero registration: CRD42024554286): *What is(are) the main developmental domain(s) evaluated by Griffiths III impaired in autism?* (RQ1); *Using Griffiths III, what is(are) the main developmental domain(s) improved after an autism-specific early intervention?* (RQ2). Methods: Six studies have been reviewed: three case–control studies, a case report study, and two studies examining the effectiveness of early autism-specific interventions. According to the study design, the methodological quality was evaluated using three standardised protocols: STROBE; JBI; CEC. Results: The results highlighted that the Language and Communication and Personal–Social–Emotional domains are the most impaired in autistic children and in those with an increased likelihood (RQ1). The results outlined that early target intervention enhanced the same domains (RQ2). Conclusions: In conclusion, the findings highlight the importance of screening not only for autism traits but also for impairments in language, communication, and socio-emotional skills. The future direction of the results is discussed.

## 1. The Child’s Developmental Profile

Concerns about a child’s development can arise from several sources (e.g., parents, educators, paediatricians). Evaluating the child’s development profile (henceforth referred to as the child’s DP) in their early developmental stage is challenging [1].

Comprehensive assessments of the child’s DP are required to identify his or her weaknesses that may necessitate targeted intervention to modify potentially atypical developmental trajectories [2,3,4]. In addition, the child’s DP assessment helps identify strengths, which practitioners can use to support the development of emerging skills or to promote new ones.

Several child-friendly and play-oriented direct gold standards for assessing the child’s DP have been standardised over time [e.g., Battelle Developmental Inventory 2nd version by Newborg [5]; Bayley Scales of Infant and Toddler Development 4th version by Bayley and Newborg [6]; Griffiths Scales by Griffiths [7]; Mullen Scales of Early Learning by Mullen [8]. The current systematic search and review aimed to devote exclusive attention to the Griffiths Scales without including or comparing other gold standard measures. The choice results from several reasons, which highlight the novelty of the review. For starters, no reviews on the third revision of the Griffiths Scales have been published, nor were there published reviews of its different versions. This means a comprehensive overview of the latest revised version of the measure is needed.

The first version of the Griffiths Scales [7] aimed to provide a developmental overview of children aged 1 month to 8 years, assessing their strengths and weaknesses by identifying both developmental precursors and indicators of atypical developmental trajectories. From its first development [7], some clarifications and amendments have been made. For instance, Griffiths II [9,10] evaluates the child’s DP from the neonatal period (1 month) to 8 years of age. Specifically, two age-specific measures have been developed: A Birth to Two Years version [9] and an extended version for children aged 2–8 years [Griffiths Mental Development Scales-Extended Revised [10].

Despite being widely used in clinical settings, differences in the organisation of subscales and scoring across these versions made it necessary to revise the measure. In response, the Association for Research in Infant and Child Development (ARICD) began a revision process in 2011, culminating in the publication of Griffiths III in 2016 [11,12].

The updated version introduced two main changes: it provides continuous developmental assessment from birth to 6 years without interruption, and it includes slightly revised subscales: *Foundations of Learning (Subscale A), Language and Communication (Subscale B), Eye and Hand Coordination (Subscale C), Personal–Social–Emotional (Subscale D), and Gross Motor (Subscale E)*. Table 1 summarises the scales, what the child’s skills are evaluated on, and some examples of items.

No revisions for scoring have been made: each item is scored as a pass (1 or +) or a failure (0 or −). Five raw scores (one for each subscale) and a general developmental quotient raw score are calculated as a sum of the passed items. Using age-specific normative tables, these raw scores provide the developmental age (DA), scaled scores, a development quotient (DQ) with a 95% confidence interval, a Statine score, and percentile values.

Griffiths III has demonstrated strong feasibility, criterion validity [13,14,15,16], and cross-cultural validity in diagnostic and follow-up settings (e.g., Italian, English, Portuguese, and Chinese versions). As such, it remains a highly suitable tool for a comprehensive assessment of a child’s developmental profile. Both earlier and revised versions of this gold-standard measure are widely used in clinical research to establish developmental baselines, especially in intervention studies (e.g., [17,18,19]).

Recently, authors of [1] and the Association for Research in Infant and Child Development [20] have stated that the revised Griffiths Scales help practitioners detect weaknesses in children with an increased likelihood of neurodevelopmental disorders, e.g., autism. Autism is a complex and pervasive disorder characterised by impairments and atypical developmental trajectories in the main developmental domains (i.e., learning, language and communication, socio-emotional skills, fine and gross motor, and independence domains, e.g., [21,22,23]). Therefore, the use of the revised version of the Griffiths Scales in autism research requires a more in-depth investigation.

## 2. The Child’s Developmental Profile in Autism

Autism is an umbrella term for several impairments [24,25], consisting of social communication and interaction deficits [26,27,28,29], socio-emotional reciprocity difficulties [30], and poor interest in peers [31]. A pattern of ritualised stereotypes and behaviours [28] and hyper- and/or hypo-reactivity to environmental sensory inputs [32] have been demonstrated. Additionally, there is evidence suggesting that autistic individuals often show reduced motor control and delayed fine motor skills [33,34,35,36].

Together, these impairments characterise autism as a complex and pervasive neurodevelopmental disorder whose onset is in early toddlerhood and continues throughout life [37,38,39], heavily impacting personal and social settings [40]. Its prevalence has risen exponentially in the past years. Indeed, the Centres for Disease Control revealed that 1 in 36 newborns [41] will be diagnosed with autism at 8–9 years.

In terms of onset, autism can manifest in one of three ways: As an early onset where impairment and deviation of social and communicative development appear as early as 12–18 months; as regressive autism characterised by a period of typical development followed by a loss of acquired abilities [42,43]; or as a developmental stagnation or plateau characterised by intact initially acquired skills that do not progress [44]. This evidence underscores the importance of early screening using reliable screeners to guide autism-specific diagnostic assessments [45,46,47,48,49,50,51,52,53,54,55,56].

On gender ratio, males are affected about four times more frequently than females [57,58]. Nevertheless, gender bias is a critically debated issue. For instance, several authors [59,60] reported that autistic females can camouflage their difficulties by showing fewer social impairments and more repetitive/restrictive behaviours, which lead to delayed diagnosis.

The assessment of a child’s developmental profile (DP) in two main contexts, diagnostic and intervention settings, is crucial. Regarding the diagnostic setting, due to its behavioural manifestation, assessment procedures for autism use parent or carer interviews, child observation, or a combination of both [61]. A multi-disciplinary team usually assesses social behaviours, verbal and non-verbal communication, adaptive and atypical behaviours, and global functioning during intensive diagnostic procedures [62,63]. This diagnostic assessment provides an evaluation not only of the severity of the autistic symptomatology but also includes an assessment of the child’s strengths and weaknesses in their DP. It is worth noting that diagnostic procedures are the critical first step for parents to gain a preliminary understanding of their child’s functioning and what lies ahead, enabling them to make decisions and plan [64]. Thus, it may not be surprising that having a comprehensive child’s DP may be a tool for the multi-disciplinary team during the communication of the diagnosis. Specifically, the comprehensive evaluation of the child’s DP may help professionals inform parents about their child’s strengths and weaknesses which, in turn, helps parents in accepting their child’s diagnosis of autism [65,66,67,68].

In intervention settings, the accurate and comprehensive evaluation of the child’s DP also served a pivotal role in planning intervention programmes [AHRQ [69]; NICE [70]]. Evidence suggests [71,72] that appropriate, early, and targeted interventions can have a substantial impact on the child’s prognosis, reducing autistic traits and improving developmental trajectories [73,74]. In the autism field, the child’s DP assessment may be a tool for professionals in defining the learning objectives. The heterogeneity among the developmental domains in autism has been demonstrated [21,22,23]. Therefore, having exhaustive information about the child’s acquired, in-progress, and unacquired abilities for each developmental domain helps professionals leverage the child’s acquired abilities that are in-progress and stimulate the emergence of the mature ones. Additionally, systematic assessments of the child’s DP may help professionals in monitoring his or her developmental trajectories. In this vein, the child’s DP assessment may be the compass for professionals to gauge the intervention activities and a tool to test their effectiveness. Professionals may check each child’s domain-specific performance to plan and/or replan the domain-specific objectives, testing the effectiveness of the intervention to improve each child’s developmental domains.

Therefore, considering the potential role served by assessing the child’s DP, providing an overview of the main studies using the revised version of the Griffiths Scales on children with an increased likelihood or those with a diagnosis of autism has important clinical and research implications.

## 3. The Current Study: The Research Questions

The present systematic search and review aimed at revising studies using Griffiths III on children with an increased likelihood or diagnosed with autism. The PICO protocol [75] was used to define the study research question(s). Specifically, we extracted studies that recruited children (low-likelihood, increased likelihood, clinical sample) aged 0–6 years (**P**articipants) and administered Griffiths III to evaluate the child’s DP (**I**ntervention). We included longitudinal and cross-sectional studies (**C**omparison) that examine the association between autistic traits and DP and/or the effectiveness of the autism-specific early intervention on the child’s DP (**O**utcome).

Based on this protocol, the main research questions addressed in this systematic search and review are as follows:

**RQ1:**  *What is(are) the main developmental domain(s) evaluated by Griffiths impaired in autism?*

**RQ2:**  *Using Griffiths III, what is(are) the main developmental domain(s) improved after an autism-specific early intervention?*

## 4. Method

The current systematic search and review applied standardised protocols in extracting and appraising eligible papers. For starters, the systematic review methodology has been pre-registered on PROSPERO (Protocol No. CRD42024554286). The PICO protocol was used to formulate the research questions. The flow diagram of the updated Preferred Reporting Items for Systematic Review and Meta-Analysis [PRISMA [76]] was followed to extract eligible papers for review. Three standardised protocols (i.e., STROBE, JBI, and CEC) appraised the methodological quality of the reviewed studies.

### 4.1. Phrase Search Syntax

Table 2 shows the detailed search strategy. A set of combined keywords using Boolean operators “AND” and “OR” according to each database syntax. Five databases have been used: SCOPUS, Web of Science, MEDLINE, PsycINFO, and CINAHL. In each database, we searched for papers with pre-defined keywords appearing in the title, abstract, subject heading, or keyword list. In addition, several filters have been applied.

### 4.2. Pilot Search Strategy and Select Eligible Papers

Table 3 summarises the set of pre-defined inclusion and exclusion criteria.

Following the 3-step PRISMA flow diagram [76], a total of 1202 records have been detected, tabulated in a .csv spreadsheet, and ordered alphabetically. During the final step, a second-hand search was performed to incorporate 2 records according to the reference lists of the reviewed studies. All duplicates (*n* = 32) were removed using automatic tools.

A final set of 1172 records was screened by two authors (AL and CM). The inter-rater agreement was calculated using a set of 50 randomly selected papers; they were independently screened by two authors (AL and CM), and their disagreements were arbitrated by a third author (FL). The inter-rater agreement for the screening procedure was excellent (Cohen’s κ = 0.93). In this step, 9 papers were excluded because of the unavailability of the full text. A total of 1156 papers were excluded according to the exclusion criteria.

Six papers [1,46,70,77,78,79] were ultimately included in the review: Three case–control studies [46,77,78], one single-case report study [1], and three [79,80] examining the effectiveness of early interventions.

**Figure 1:**
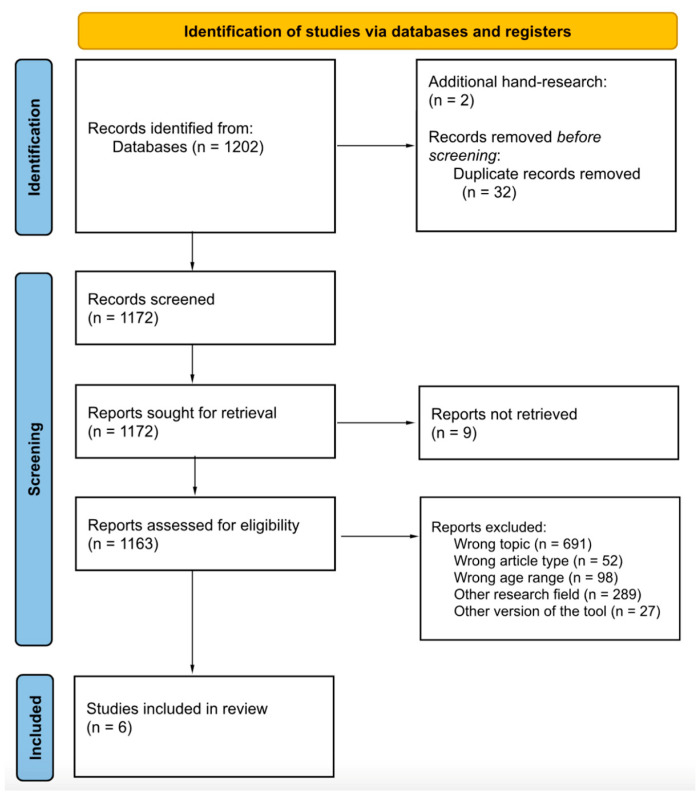
The PRISMA flow diagram.

The PRISMA flow diagram [76] maps the entire selection process (Figure 1).

### 4.3. Methodological Quality Appraisal of Reviewed Studies

According to the study design of the reviewed studies, three protocols have been used to appraise their methodological quality. The 22-item STROBE checklist [81] was applied to assess the methodology quality of the three case–control studies. The checklist covers all sections of the article: title and abstract (item 1), introduction (items 2 and 3), methods (items 4–12), results (items 13–17), discussion section (items 18–21), and additional information (item 22 on funding). For some items (i.e., 8, 13, 14, 15), information should be provided separately for exposed and unexposed groups. Each item is scored as a pass (1) or fail (0); if not applicable, NA is assigned. A maximum possible score of 40 is calculated as the sum of all items. The study’s methodological quality is calculated as the percentage of passed items. Four categories have been conceived: poor (0–25%), fair (26–50%), good (51–75%), and excellent (76–100%).

To assess the methodological quality of the single-case report study, the 8-item JBI’s Critical Appraisal Tools Assist [82] was applied. It covers the following sections: participants’ demographic/clinical characteristics and history (items 1–3), methods (item 4), intervention and post-intervention procedures (items 5–6), description of adverse or unexpected events (item 7), and implications (item 8). Each item is scored as a pass (1) or fail (0); if not applicable, NA is assigned. A maximum possible score is calculated as the sum of eligible items. The study’s methodological quality is calculated as the percentage of passed items. Four categories have been conceived: poor (0–25%), fair (26–50%), good (51–75%), and excellent (76–100%).

Finally, to appraise the methodological quality of the intervention studies, the 28-item Council of Exceptional Children’s Evidence-based Practice Standards [CEC [83]] was used. All sections of the article are covered by the checklist: context and setting (item 1.1), participants (items 2.1 and 2.2), intervention agent (items 3.1 and 3.2), description of practice (items 4.1 and 4.2), implementation fidelity (items 5.1–5.3), internal validity (items 6.1–6.9), outcome measures/dependent variables (items 7.1–7.6), data analysis (item 8.1–8.3). Out of these, 18 quality indicators pertain to between-group and single-case research designs, 6 are relevant exclusively to between-group designs, and 4 exclusively to single-case research. Each item is scored as a pass (1) or fail (0); if not applicable, NA is assigned. A maximum possible score is calculated as the sum of eligible items. The study’s methodological quality is calculated as the percentage of passed items. Four categories have been conceived: poor (0–25%), fair (26–50%), good (51–75%), and excellent (76–100%).

## 5. Results

### 5.1. Summary of the Reviewed Studies

The main information of each study has been tabulated (Table 4), and the papers are presented alphabetically and according to the study category (case–control vs. case-report vs. intervention). Specifically, the country in which the study was conducted, the study design (cross-sectional vs. longitudinal), the sample characteristics (i.e., sample size, gender distribution, mean age, standard deviations, and age range), the measures administered, the study purpose(s), and the relevant results were summarised. In addition, information about the intervention (i.e., duration, waves, and child’s evaluations) has been included. A narrative approach is used to resume each study.

The three case–control studies [46,77,78] and the two studies evaluating the effectiveness of early interventions [79,80] have been conducted in Italy. The case report did not report this information [1]. Only one case–control study is longitudinal [46], whereas the other ones are cross-sectional.

In the following paragraph, a study-specific discussion is provided.

#### 5.1.1. Case–Control and Case Report Studies

The study by Cirnigliaro et al. [77] matched for developmental age three groups of 18–48-month toddlers: The autistic group (ASD group), the global developmental delay group (DD group), and the typically developing one (TD group). The main study’s purpose is to develop (Study 1) and test the diagnostic accuracy and criterion validity (Study 2) of a novel observational and interactive level 2 screener for autism, i.e., Developmental Autism Early Screening (DAES). The measure is based on the critical scores reached by autistic toddlers using Griffiths Scales III. Specifically, on a total of 87 toddlers (*n* = 26 children in each group), multiple comparison tests showed that the ASD group achieved lower scores than DD and TD in the B subscale (Language and Communication); furthermore, the ASD group performed worse than DD and TD groups on the D (Personal–Social–Emotional) subscale. Following these results, authors considered these two subscales as the most sensitive in capturing differences in developmental profiles among the ASD and DD/TD groups. The single-item analysis showed the 36 items of the B and D subscales, which differed significantly among groups. As a consequence, the authors suggested that they represent the most predictive items in detecting children with an increased likelihood of autism. The diagnostic accuracy of the DAES, as well as the correlations between the novel screener and the gold standard for the autism diagnosis [i.e., the Autism Diagnostic Observational Schedule—2nd version [85]], have been assessed (Study 2). In addition, the cut-off of 12.5 enabled differentiation between children of the ASD group from those of the DD and TD groups, highlighting good accuracy (sensitivity: 93%; specificity: 98.4%; Positive Predictive Value: 96.3%, Negative Predictive Value: 96.9%). Regarding the criterion validity, the DAES was positively correlated with Autism Diagnostic Observational Schedule-2 scores (i.e., symptom severity, socio-affective, and repetitive restricted behaviours scores). This means that the lower the quotient in the B and D subscales, the higher the ASD symptoms. In sum, the novel screener probes several skills, such as listening, attention and communicative intent, expressive communication, receptive language development, social-emotional reciprocity, social communication and interaction, play, social cognition, joint attention, self-awareness, emotional understanding and expression, and empathy as the main features in detecting children with an increased likelihood of autism.

The study by Levante et al. [46] on 11–24-month-old toddlers tested the psychometric properties [i.e., criterion validity with gold standard measures; Autism Diagnostic Observational Schedule—2nd version [85]] of two screening instruments for autism, i.e., the First Year Inventory (FYI [86]) and the Quantitative-CHecklist for Autism in Toddlers (Q-CHAT [56]). This 3-wave longitudinal study involved toddlers screened with an increased likelihood of autism in the general population. The first wave (T1) enrolled 657 children aged 11–13 months who were screened using the FYI. The second wave (T2) involved 545 children aged 18–21 months who were screened with the Q-CHAT. The third wave (T3) consisted of the diagnostic assessment of these at-risk groups, comparing 12 children with a high FYI score and 11 with a high Q-CHAT score to 15 typically developing (TD) toddlers. During T3, Griffiths III was administered to assess the developmental profiles of all children. The results showed significant differences between both groups with an increased likelihood of autism and the TD across all subscales except for the gross motor (subscale E). Notably, the poorest scores were observed on the Language and Communication (subscale B) and Personal–Social–Emotional (subscale D) scales for both groups with an increased likelihood of autism. The study also found that the severity of autistic symptoms was negatively associated with the developmental profile from early months of life. Specifically, higher autistic symptoms were associated with lower performance on subscale D when increased likelihood was detected at 11–13 months, and these symptoms were correlated with subscales B, C, and D during the second year of life. The results confirmed the criterion validity of the FYI and Q-CHAT. Children identified with an increased likelihood of autism at 11–13 months and 18–21 months exhibited restricted global functioning compared to non-autistic children.

Finally, the study by Taddei et al. [78] aimed to compare the developmental profiles of two groups of preschool children: One group co-diagnosed with autism and developmental disabilities (ASD + DD), and another one with developmental disabilities (DDs) solely. The study used Griffiths III normative tables for the Italian population to compare the scores between autistic and non-autistic samples. Both the ASD + DD and DD groups exhibited lower age-equivalent scores compared to their chronological age across all Griffiths III developmental subscales. While the mean difference in age-equivalent scores between the two groups across the five scales was negligible, the developmental profiles revealed distinct patterns. The DD group demonstrated a homogeneous developmental profile with similar levels of delay in all domains. In contrast, the ASD + DD group showed weaknesses, particularly in the Language and Communication (subscale B) and Personal–Social–Emotional (subscale D) subscales.

The case report study by Jansen et al. [1] on an autistic 6-year-, 4-month-old boy with autism aimed to assess the effectiveness of the Griffiths III scales in supporting the diagnosis of autism and possible Attention Deficit/Hyperactivity Disorder (ADHD). The study goal was to describe the baseline of the child before the intervention. The child’s developmental profile, evaluated by using the Griffiths III scales, revealed a low developmental age (DA) in all subscales. Specifically, the child fell into the extremely low range on subscale A (Learning Foundation; DA: 55 months; DQ < 50), subscale B (Language and Communication; DA: 50 months; DQ ≤ 50), and subscale D (Personal–Social–Emotional; DA: 53 months; DQ < 50). Additionally, the child fell into the borderline range on C (Eye and Hand Coordination) and E (Gross motor) subscales, suggesting some challenges in these domains, but to a lesser extent. Although other measures have been administered to the child, no associations with Griffiths III were computed.

#### 5.1.2. Intervention Studies

Two papers [79,80] investigating the effectiveness of early intervention for autism have been reviewed. Both studies administered Griffiths III to evaluate whether the children’s DP changed because of the intervention.

Colombi et al. [80] examined the effectiveness of the Early Start Denver Model (ESDM) on a group of autistic children. The 6-month longitudinal study compared 22 autistic children who received the 6 h/week ESDM intervention (ESDM group) to a group of 70 peers who received Treatment as Usual (TAU group) for 5.2 h/week. All children were assessed before the intervention (baseline) and after 3 and 6 months of intervention using standardised instruments, i.e., the Griffiths III scales, the Vineland Adaptive Behaviour Scale—2nd Edition [91], and the Autism Diagnostic Observation Schedule—2nd version [85]. Overall, results reported that after 3 and 6 months of intervention, all children (i.e., ESDM and TAU groups) showed a statistically significant improvement in all domains (for details, see [80]). On children’s developmental profile in Griffiths III, the results showed that—after 3 and 6 months of target intervention—the ESDM group showed an improvement in B (Language and Communication) and D (Personal–Social–Emotional) subscales compared to the TAU group. After 6 months, results highlighted that the ESDM group’s performance in the C (Hand and Eye Coordination) and E (Gross Motor) subscales was greater than the TAU group.

The case report by Colombi et al. [79] aimed to evaluate the impact of early intervention on a 6-month-old male toddler who showed an increased likelihood of autism. Specifically, the study aimed to evaluate the effectiveness of a parent-mediated intervention based on the ESDM assumptions in improving the child’s developmental profile. The child’s DP was evaluated at 8, 14, 19, and 32 months of age. As reported by the authors, there has been a statistically significant improvement over time in the child’s performance in all developmental domains evaluated by Griffiths III. Indeed, the child’s age equivalent was close to the chronological one after the intervention. This means that the child’s performance reached a consistent increase, as well as the developmental quotient in all Griffiths III subscales. It is worth noting that the child’s age equivalent in B (Language and Communication) and D (Personal–Social–Emotional) subscales were the most increased, as well as the developmental quotients.

### 5.2. Methodology Quality Appraisal

#### 5.2.1. Quality Appraisal: STROBE Statement

The methodological quality of the study by Cirnigliaro et al. [77] is excellent (the study passed 80% of the protocol items). In sum, the study provided comprehensive information across all sections of the article. The main flaws that the protocol highlighted include the lack of some information in the title and abstract (i.e., indicating the study’s design with a commonly used term) and the lack of description of possible sources of bias. The authors did not report some information regarding the statistical analysis and sample; that is, how missing data have been handled and the number of participants with missing data. Nevertheless, it is worth noting that the authors administered all measures face-to-face on the clinical sample. Thus, it may be reasonable to assume that there are no missing data. The generalisability of the results was not discussed. Despite these limitations, several strengths have to be highlighted: A clear theoretical background supporting the aims and hypotheses was provided; the study design and eligibility criteria for recruiting the sample, as well as the study results, were well described. The discussion was well organised, and the interpretation of the results was based on data.

The methodological quality of the study by Levante et al. [46] is excellent (the study passed 80% of the protocol items). In sum, the study provided comprehensive information across all sections of the article. The main flaws acknowledged were the lack of a detailed description of potential sources of bias; some information regarding the statistical analysis and participants was missing; that is, how missing data have been handled and the number of participants with missing data. Nevertheless, it is worth noting that the authors administered all measures face-to-face on the clinical sample. Thus, it may be reasonable to assume that there are no missing data. Despite these flaws, the study’s design and methodology were rigorous. The longitudinal nature of the research enhances its generalisability, supporting a more comprehensive understanding of early risky behaviours for autism detection. The administration of screening measures on a large sample size may support the reliability of its results and their relevance for clinical settings.

The methodological quality of the study by Taddei et al. [78] is excellent (the study passed 80% of the protocol items). In brief, the study provided comprehensive information across all sections of the article. The flaws acknowledged were the lack of description of possible sources of bias, missing information regarding statistical analyses (i.e., handling of missing data and details on sensitivity analyses), and results. Nevertheless, these limitations did not affect the overall methodological quality of the study. Indeed, the study had a rigorous methodology and administered standardised tools, which support the interpretation of its results. The application of comprehensive statistical methods further underlined the reliability of the results. Additionally, the study presented its main results clearly, providing valuable insights for professionals engaged with autistic children.

The rating of each paper by the STROBE protocol is shown in Table S1 (Supplementary Materials).

#### 5.2.2. Quality Appraisal: JBI Critical Appraisal Tool

The methodological quality of the single-case report [1] is excellent (the study passed 83.3% of the protocol items). The flaws acknowledged were the lack of information regarding the identification/description of adverse events (harms) or unanticipated events. Despite this, the study reported detailed, comprehensive descriptions of the patient’s history, clinical condition, diagnostic methods, treatment procedures, and outcomes, contributing meaningfully to the study’s overall quality. The evaluation of the paper using the JBI protocol is shown in Table S2 (Supplementary Materials).

#### 5.2.3. Quality Appraisal: CEC Protocol

The methodological quality of the study by Colombi et al. [80] is good (the study passed 75% of the protocol items). The flaws acknowledged were the lack of information about the materials (e.g., manipulatives, timers, cues, toys) used during the intervention, as well as the lack of information about the internal validity (i.e., attrition), internal reliability and effects size coefficients. Nevertheless, the study had a rigorous methodology, clear descriptions, and valuable results. Specifically, the study offered a rationale for the study’s purposes and a clear description of the intervention setting, facilitating the understanding of the environment where the intervention took place. Detailed information on participants was provided, e.g., demographic characteristics and inclusion/exclusion criteria for recruiting children. The roles and qualifications of the intervention agents were widely described. The intervention procedures and practices were clearly outlined, supporting the replicability of the study. A robust statistical analysis plan was reported, and the outcomes were in line with the study’s purposes.

The methodological quality of the study by Colombi et al. [79] is excellent (the study passed 79.2% of the protocol items). The flaws acknowledged were the lack of information on the materials used during the intervention, as well as the lack of assessment of fidelity and internal reliability. Nevertheless, a detailed overview of the context and setting, as well as comprehensive participant (i.e., demographic characteristics and inclusion/exclusion criteria for recruiting sample) information was provided. The roles and qualifications of the intervention agents were documented. The intervention procedures were widely explained, supporting the replication of the research. The statistical analyses and the outcomes were in line with the study’s purposes. The evaluation of the papers using the CEC protocol is shown in Table S3 (Supplementary Materials).

## 6. Discussion

The current systematic search and review devoted attention to studies using Griffiths III; that is, the revised version of the child-friendly and play-oriented direct gold measures of the child’s developmental profile (DP). Following evidence [77,94,95] supporting the measure enables an evaluation of the 0–72-month-old child’s DP with an increased likelihood of autism or is diagnosed with autism; the current review extracted and reviewed papers recruiting from this population.

Although Griffiths III has been cross-culturally validated, the measure is not widely administered in research settings. Indeed, six papers have been reviewed and appraised using study design-specific standardised protocols. These protocols appraised their methodological quality, which ranged from good to excellent. In addition, the appraisal highlighted that the main study flaws (e.g., missing data handling or lack of some sample information) are shared among the reviewed studies; nevertheless, they did not affect their methodological quality.

Based on the high quality of the reviewed studies, two research questions were addressed: What is(are) the main developmental domain(s) evaluated by Griffiths III impaired in autism? (RQ1). Using Griffiths III, what is(are) the main developmental domain(s) improved after an autism-specific early intervention? (RQ2).

Regarding RQ1, we reviewed both case–control studies [46,77,78] and a single-case report [1]. Overall, studies highlighted those children with an increased likelihood of autism, and those with autism showed weaknesses in all domains evaluated using Griffiths III. For instance, one study [46] reported that children aged 11–13 months and 18–21 months with an increased likelihood of autism showed a lower score in all DP scales (except for the E subscale—Gross Motor) than TD peers. Another study [78] outlined that the autistic group showed the lowest scores in B (Language and Communication) and D (Personal–Social–Emotional) subscales. Conversely, the DP of children with developmental disorders (without autism) is impaired but homogeneous among the domains.

Another concern arising from reviewed studies is in regard to the serious weaknesses of subscales B (Language and Communication) and D (Personal–Social–Emotional) of children with an increased likelihood of autism and diagnosed ones. These limits emerged for autistic children in comparison with TD peers [46,77] and DD children [77,78]. In this vein, although only two studies [46,77] explored the association between DP domains and autistic symptoms, results spoke about a negative correlation with the B (Language and Communication) and D (Personal–Social–Emotional) subscales. This means that the higher the autistic symptoms, the lower the scores in language and communication (B subscale) and personal, social, and emotional domains. In addition, the reviewed longitudinal study [46] reported that the association between the autistic symptoms and subscale D is strong when children were 11–13 months old, whereas the association between autistic symptoms and subscales B and C (Eyes and Hand Coordination) is strong when children were 18–21 months old.

Overall, a reflection on the methodological flaws of the reviewed studies arises. Although promising results were found, the flaws related to the sample size, the lack of sex-specific analyses, and the single-country bias were highlighted, requiring further investigations. For instance, the small sample size may limit the generalisability of the results; nevertheless, the difficulty in recruiting vulnerable populations such as the autistic population has been demonstrated [96,97]. In addition, considering the sex ratio discrepancy in autism, sex-specific analyses would be mandatory in expanding knowledge on the topic. Although it is not closely related to the autistic symptomatology, further studies should be designed to explore the topic in the global north and south.

Overall, there is a non-conforming snapshot of DP for children with an increased likelihood and those with a diagnosis of autism. It is worth pointing out that domains B and D are the ones to be monitored most in toddlerhood. Delays in the development of communication and language are among the greatest parental concerns [98] between 12 and 24 months. There were also parental concerns regarding the development of autonomy, social skills, and emotional reciprocity are reported by parents when children are 24 months, which is the developmental stage in which these skills are in progress or already acquired by the child [99].

The second research question (RQ2) investigated whether Griffiths III provides an evaluation of the change in a child’s DP due to early autistic-specific interventions, especially for one that involves all domains simultaneously. The results of the two reviewed studies [79,80] showed that early intervention improved the B (Language and Communication) and D (Personal–Social–Emotional) subscales after only 9 weeks of intervention [80]. In addition, the single-case report [79] showed that the equivalent age and development quotient of these two scales improved more than the other development domains. The C (Eye and Hand Coordination) and E (Gross Motor) subscales also showed enhancements after only 6 months of intervention [80].

Overall, the results paved the way for two close reflections on the need for early screening and intervention for developmental delay. On one side, all reviewed studies reported that the Language and Communication (scale B) and the Personal–Social–Emotional (scale D) domains of the child’s DP are the ones most impaired. These results confirmed parental concerns [98] regarding the child’s development and support the request to health care professionals not to underestimate them. On the other side, the same domains have been identified as the one most improved after an early intervention. This supports the need to assess the specific domains as early as possible to modify the children’s atypical developmental trajectory.

In conclusion, these results support the mandatory monitoring of a child’s language (e.g., speech) and communication development (e.g., gestures, pointing, eye contact) as well as of personal, social, and emotional skills (e.g., social smile, anticipatory movements, adult orientation, social openings) in children under 24 months. This would allow monitoring of the child’s development to diagnose as early as possible and plan the early intervention.

## 7. Strengths, Limitations, and Future Directions

The main strengths of the current review include providing the first overview of the evidence using the revised version of the Griffiths Scales on children with an increased likelihood of autism and autistic children, as well as the appraisal of the methodological quality of the reviewed study following standardised protocols, from which the main information was extracted. Furthermore, information on what domain(s) is(are) mainly impaired in children on the spectrum was provided.

Review limitations are related to those of the reviewed studies. For starters, although Griffiths III has been translated into different countries (e.g., English, Portuguese, and Chinese versions; see the publisher’s website for details), most of the reviewed studies have been carried out in Italy. Thus, the single-country bias leads to interpreting the results carefully. In addition, a paucity of longitudinal studies emerged. Due to the small/moderate sample size recruited by the reviewed studies, generalisability is limited. Additionally, several sample characteristics were missing, as is often the case in autism research (e.g., [100]). Due to the inclusion of two papers where the current review’s authors are coauthors, the selection bias is met. Nevertheless, the application of a standardised protocol appraising the methodological quality supported the suitability of the papers for the review’s purposes. In addition, to manage the bias, the current review’s author, who is not a coauthor in the reviewed papers, was asked to appraise and critically review the papers. Lastly, and unfortunately, nine studies have not been reviewed due to the unavailability of the full text. Although their abstracts provided important insights into the hypothetical eligibility of these papers for review purposes, their inclusion in this review is not certain due to the predefined peculiar criteria. Nevertheless, careful interpretation of the review’s conclusion is advisable.

The current systematic search and review paves the way for future directions. Further longitudinal as well as cross-sectional studies are required to support the results summarised in this review, discussing the cultural sample characteristics. Though few, all the reviewed studies point in the same direction; that is, the notable impairment in the Language and Communication (scale B) and in the Personal–Social–Emotional (scale D) domains. Longitudinal studies could compare different groups of children [e.g., high (i.e., sibling) vs. low-risk or ASD group vs. DD group (or other neurodevelopmental disorders)] to investigate whether the domains of the developmental profile may change over time under different conditions (experimental vs. control) and following different interventions.

In addition, the results of this review suggested the need to define more comprehensive screening procedures for the general population: This means administering standardised questionnaires assessing not only the autistic traits but also language and communication skills, as well as the socio-emotional traits. These screening procedures are strongly recommended by the American Academy of Paediatrics, especially for children under 24 months of age. This preliminary screening may have a positive cascade effect on healthcare providers who could have information on the child’s competencies (in the form of a parent report questionnaire) to plan out a diagnostic assessment. The paediatricians who may suggest and support the family in early screening procedures could be relevant. Given the importance of parental trust in paediatricians [101] in the prompt delivery of information about their child’s health status, it may be pertinent to investigate the quality of this dyadic relationship.

Note: To follow a rigorous methodological quality appraisal of the reviewed papers, the papers by Levante et al. [46] and Colombi et al. [79] were appraised by the au-thor (CM) not involved in the paper.

## Figures and Tables

**Table 1:** Griffiths III domains, their brief description, and an example item to evaluate the domains.

Domains (Subscale)	Skills Assessed	Item Example
*Foundations of Learning* (subscale A)	Assess the child’s ability to learn (e.g., including attention, problem-solving abilities, sequential reasoning, processing speed, visuospatial skills, and memory).	Ask the child to build a bridge with blocks after the demonstration.
*Language and Communication* (subscale B)	Evaluate the child’s receptive and expressive language and communication abilities.	Ask the child to name objects and pictures, define objects by their use, and follow instructions.
*Eye and Hand Coordination* (subscale C)	Assess the child’s visual perception and fine motor skills.	Ask the child to stack or build a tower with blocks.
*Personal–Social–Emotional* (subscale D)	Evaluate the child’s ability to adapt, his or her independence, and early socio-emotional abilities. Assess the child’s imitation, joint attention, emotional recognition, and empathy.	The child identifies body parts, participates in group games, and pronounces their name.
*Gross Motor* (subscale E)	Assess the child’s early development of postural control, gross body coordination, balance, and visual–spatial coordination.	Ask the child to run, jump, or walk in a straight line.

**Table 2:** Search strategy applied.

Search Date	Search Strategy	Filters	Sources
5 June 2024	“autis*” OR “*ASD*” OR “*autism spectrum condition*” OR “*ASC*” OR “develop* disorder” OR “neurodevelop* disorder” OR “*pervasive disorder*” OR “*autistic spectrum*” OR “*autism spectrum disorder*”	*Subject area*: psychology; social sciences; health professions; multidisciplinary; neurosciences.	SCOPUS
	AND	*Document type*: peer-reviewed articles.	Web of Science
	“diagnos*” OR “early diagnos*” OR “assess*” OR “evaluat*” OR “measur*”	*Source type*: academic journals.	MEDLINE
	AND	*Language*: English.	PsycINFO
	“*Griffiths*” OR “*Griffiths scales of child development*” OR “*Griffiths-III*” OR “*Griffiths III*” OR “*GSCD*” OR “*GSCD-III*” OR “*Griffiths development scale*” OR “*Griffiths 3rd*” OR “*Griffiths 3rd edition*”	*Age group*: children aged 0–6 years.	CINAHL

Note: * indicates the use of truncation to include all relevant word variants in the search.

**Table 3:** Inclusion and exclusion criteria.

Inclusion Criteria	Exclusion Criteria
(1) Empirical studies (i.e., cross-sectional, case–control study, intervention studies). (2) Studies administering Griffiths III. (3) Children with a low likelihood or increased likelihood for autism; autistic children. (4) Children aged ≥ 0 and ≤6 years. (5) Papers published between 1 January 2016 and 6 May 2024.	(1) Studies using previous versions of the Griffiths Scales. (2) Participants aged ≥ 6 years. (3) Papers that did not report the participants’ age or mean scores of assessment measures. (4) Non-empirical studies (e.g., dissertations, conference abstracts and/or papers, editorials, opinions, commentaries, recommendations, letters, books, and book chapters; other systematic, non-systematic reviews, and meta-analyses).

**Table 4:** Main descriptive information of each included study.

	**References [Study Authors (Year)],**	**Country**	**Study Design (Cross-Sectional vs. Longitudinal)**	**Sample Characteristics: Sample Size, Mean Age and Standard Deviation, Age Range**		**Measures**	**Study Purpose(s)**	**Relevant Results**
**Case–Control studies**								
**1**	Cirnigliaro et al., [77]	Italy	Cross-sectional study	Total sample age range: 18–48 months. Study 1 N = 78 children. Specifically: ASD group: N = 26 children with ASD (males: 76.92%). M(SD) = 39.46 (12.48) months. DD group: N = 26 children with DD (males: 84.62%). M(SD) = 34.07 (7.88) months. TD group: N = 26 TD children (males: 69.23%). M(SD) = 26.38 (7.48) months. Study 2 N = 219 children. Specifically: ASD group: N = 57 children with ASD (males: 75%). M(SD) = 32 (8.00) months. DD group: N = 61 children with DD (males: 82%). M(SD) = 34 (7.5) months. TD group: N = 101 TD children (males: 51%). M(SD) = 22 (8.5) months.		Child’s developmental profile: Griffith Scales of Child Development (Griffiths III [11]). Child’s risky behaviours for autism: Developmental Autism Early Screening (DAES [77]). Autistic symptoms: Autism Diagnostic Interview-Revised (ADI-R [84]). Autism Diagnostic Observation Schedule, second edition (ADOS-2 [85]).	Study 1: Development of a screener for detecting at-risk children for autism according to the Griffiths Scales of Child Development III. Study 2: Assessment of the reliability and validity of the screener.	Study 1: The B (Language and Communication) and D (Personal–Social–Emotional) subscales of Griffiths III were the most sensitive in capturing differences between groups: differences between the ASD and DD/TD were found on the B (Language and Communication) and D (Personal–Social–Emotional) subscale. Study 2: Analyses supported the diagnostic accuracy and criterion validity. Gender differences were not computed.
**2**	Levante et al., [46]	Italy	Longitudinal study	T1 N = 12 children (males: 50%). M(SD) = 12.5 (0.54) months. Age range: 11–13 months. T2 N = 11 children (males: 72%). M(SD) = 19.6 (3.6) months. Age range: 18–21 months. T3 N = 16 children (males. 66%). M(SD) = 12.11 (0.92) months. Age range: n.s.		Child’s developmental profile: Griffith Scales of Child Development (Griffiths III [11]). Child’s risky behaviours for autism: First Year Inventory (FYI [86]). Quantitative-CHecklist for Autism in Toddlers (Q-CHAT [56]). Autistic symptoms: Autism Diagnostic Observation Schedule, second edition (ADOS-2 [85]). Child’ internalising/externalising symptoms: Child Behaviour Checklist (CBCL [87]).	Examine the criterion validity of two screeners, i.e., FYI and the Q-CHAT.	The criterion validity is supported by results. Preliminary accuracy data have been provided. Differences were found between both at-risk groups and the typically developing (TD) group across all subscales except gross motor. At-risk groups had the lowest scores in Griffiths III subscales B (Language and Communication) and D (Personal–Social–Emotional). Furthermore, the severity of ASD symptoms negatively correlated with DP (with subscale D at 11–13 months and with subscales B, C, and D during the second year of life). Gender differences were not computed.
**3**	Taddei et al., [78]	Italy	Cross-sectional study	Total sample age range: 6–68 months. N = 74 children. Specifically: ASD + DD group: N = 39 children with ASD + DD (males = 82.05%). M(SD) = 42.6 (15.5) months. DD group: N = 35 children with DD (males = 68.57%). M(SD) = 31.7 (16.5) months.		Child’s developmental profile: Griffith Scales of Child Development (Griffiths III [11]).	Identify developmental profiles associated with Autism Spectrum Disorder and global developmental delay in preschool-aged Italian children using Griffiths III.	Both groups showed developmental delays across all subscales according to their chronological age. Children with ASD + DD showed low scores across all subscales and the lowest score for B (Language and Communication) and D (Personal–Social–Emotional) subscales. The DD group exhibited a consistent delay across all subscales. Gender differences were not computed.
**Case report study**								
**4**	Jansen et al., [1]	Not specified	Cross-sectional study	N = 1 child with ASD (male). Age: 6 years and 4 months.		Child’s developmental profile: Griffith Scales of Child Development (Griffiths III [11]). Autistic symptoms: Childhood Autism Rating Scale (CARS [88]). Child’s hyperactivity and attention deficits: Conners 3-Parent and Teacher Surveys, Long Form [89]. Child’s cognitive profile: Goodenough–Harris Draw-a-Person Intellectual Ability Test (DAP: IQ [90]).	Use Griffiths III to clarify a diagnosis of Autism Spectrum Disorder and possible Attention Deficit/Hyperactivity Disorder in a 6-year-old male child.	The child exhibited a low developmental age (DA) across all subscales, with extremely low scores in subscales A (Learning Foundation), B (Language and Communication), and D (Personal–Social–Emotional). For subscales C (Eye and Hand Coordination) and E (Gross Motor), the child fell within the borderline range.
**Intervention studies**								
	**References [Study Authors (Year)],**	**Country**	**Study Design (Cross-Sectional vs. Longitudinal)**	**Description of the Intervention**	**Sample Characteristics: Sample Size, Mean Age and Standard Deviation, Age Range**	**Measures**	**Study Purpose(s)**	**Relevant Results**
**5**	Colombi et al., [80]	Italy	Pre- and post- longitudinal study	Intervention duration: 6-months intervention Intervention wave: 6 h/week Child’s evaluation: baseline, after 3 months, and post-test (after 6 months).	Experimental group (ESDM intervention group) N = 22 children with ASD (gender distribution: n.s.) M(SD) = 31.1 (8.0) months. Control group (TAU intervention group) N = 70 children with ASD (gender distribution: n.s.) M(SD) = 35.2 (7.6) months. Total sample age range: 18–48 months.	Child’s developmental profile: Griffith Scales of Child Development (Griffiths III [11]). Autistic symptoms: Autism Diagnostic Observation Schedule, Second Edition (ADOS-2 [85]). Child’s adaptive behaviours: Vineland Adaptive Behaviour Scales-2 (VABS-II [91]).	Focusing on the children’s DP and their adaptive behaviours, evaluating the effectiveness of the Early Start Denver Model (ESDM) intervention by comparing a group of children who received ESDM to a group of children who received treatment as usual.	Children in both groups improved in cognitive, adaptive, and social skills after 3 and 6 months of treatment. However, the ESDM group achieved greater improvements in cognitive and social skills after 3 and 6 months of treatment than the control group. The ESDM group achieved greater improvements in adaptive skills than the control group after 3 months of treatment. Results on children’s developmental profiles indicated that, after 3 and 6 months of intervention, the ESDM group exhibited increased scores in subscales B (Language and Communication) and D (Personal–Social–Emotional) compared to the TAU group. Furthermore, after 6 months, the ESDM group also showed improved scores in subscales C (Hand and Eye Coordination) and E (Gross Motor) compared to the TAU group. Gender differences were not computed.
**6**	Colombi et al., [79]	Italy	Single-case report longitudinal study	Intervention duration: Child aged 6–8 months: 2 h/week (P-ESDM intervention). Child aged 9 months: 30 min twice a day (ESDM intervention). Child aged 11–20 months: 1 h/week (P-ESDM intervention) and 2 h/week (ESDM intervention). Child aged 23–32 months: 1 h/week (ESDM intervention). Child’s evaluation: 6–8 months, 14, 19, and 32 months.	N = 1 child at risk of ASD (male). Age: 6 months.	Child’s developmental profile: Griffith Scales of Child Development (Griffiths III [11]). Autistic symptoms: Autism Diagnostic Observation Schedule, Second Edition (ADOS-2 [85]). Child’s adaptive behaviours: Vineland Adaptive Behaviour Scales-2 (VABS-II [91]). Child’s risky behaviours for autism: Social Attention and Communication Surveillance-Revised (SACS-R [92,93]). Child’s neurological evaluation: EEG/MRI.	To report the case of a child showing early signs of ASD in the first few months of life. The child received parent-mediated preventive intervention based on the Infant Start model, an adaptation of the Early Start Denver (ESDM) model. The progression of the child’s developmental profile was investigated.	Repetitive evaluations showed progressive improvements in developmental level and ASD symptoms. The child showed improvement over time in all developmental domains evaluated by Griffiths III. Specifically, B (Language and Communication) and D (Personal–Social–Emotional) subscales were the most increased. The child’s age equivalent was close to the chronological one at the end of the intervention.

Note: M(SD): Mean (Standard Deviation); ASD: Autism Spectrum Disorder; DD: Developmental Disability; TD: Typically Developing; DP: Developmental Profile; P-ESDM: Parent-mediated Early Start Denver Model—Intervention.

## Supplementary Materials

The following supporting information can be downloaded at: https://www.mdpi.com/article/10.3390/brainsci15050506/s1. Table S1. STROBE items for case–control studies. Table S2. JBI items for case report studies. Table S3. CEC items for intervention studies.
