# Supplementary material for: The Use of Griffiths III in the Appraisal of the Developmental Profile in Autism: A Systematic Search and Review
Source: Brain Sci. 2025 May 15;15(5):506. doi: 10.3390/brainsci15050506 (PMC12110223; doi:10.3390/brainsci15050506)
Supplement: Supplementary file 1 [file brainsci-15-00506-s001.zip › brainsci-3606682-supplementary.pdf]

Table S1. STROBE items for case-control studies.

	Statement—Checklist of items that should be included in reports of case-control studies	*Maximum possible score*	Cirnigliano et al., (77)			Levante et al., (46)			Taddei et al., (78)		
			**Main document**	**Case**	**Control**	**Main document**	**Case**	**Control**	**Main document**	**Case**	**Control**
Title and abstract	1a	1	0			1			1		
	1b	1	1			1			1		
Introduction											
Background/rationale	2	1	1			1			1		
Objective	3	1	1			1			1		
Method											
Study design	4	1	1			1			1		
Setting	5	1	1			1			1		
Participants	6a	1	1			1			1		
	6b	1	1			1			1		
Variables	7	1	1			1			1		
Data sources/measurement	8*	2		1	1		1	1		1	1
Bias	9	1	0			0			0		
Study size	10	1	1			1			1		
Quantitaive variables	11	1	1			1			1		
Statistical method	12a	1	1			1			1		
	12b	1	1			1			1		
	12c	1	0			0			0		
	12d	1	1			1			1		
	12e	1	1			1			0		

Results											
Participants	13a*	2		1	1		1	1		1	1
	13b*	2		1	1		0	0		1	1
	13c*	2		1	1		1	1		0	0
Descriptive data	14a*	2		1	1		1	1		1	1
	14b*	2		0	0		0	0		0	0
Outcome data	15*	2		1	1		1	1		1	1
Main results	16a	1	1			1			1		
	16b	1	1			1			1		
	16c	1	0			0			0		
Other analyses	17	1	1			1			1		
Discussion											
Key results	18	1	1			1			1		
Limitation	19	1	1			1			1		
Interpretation	20	1	1			1			1		
Generalisability	21	1	0			1			1		
Other information											
Funding	22	1	NA			NA			1		
	Total	40	32			32			32		
			80%			80%			80%		

* Give information separately for exposed and unexposed groups. NA: not applicable.

Table S2. JBI items for case report studies.

JBI Checklist	*Maximum possible Score*	Jansen et al., (1)
Item 1. Were the patient's demographic characteristics clearly described?	1	1
Item 2. Was the patient's history clearly described and presented as a timeline?	1	1
Item 3. Was the current clinical condition of the patient on presentation clearly described?	1	1
Item 4. Were diagnostic tests or assessment methods and the results clearly described?	1	1
Item 5. Was the intervention(s) or treatment procedure(s) clearly described?	NA	NA
Item 6. Was the post-intervention clinical condition clearly described?	NA	NA
Item 7. Identification/description of adverse events (harms) or unanticipated events	1	0
Item 8. Does the case report provide takeaway lessons?	1	1
Total	6	5
		62.5%

Table S3. CEC items for intervention studies.

	S Item	*Council of Exceptional Children's Evidence-based Practice Standards (CEC) (Cook et al., 2015)*	*Maximum possible Score*	Colombi et al., (80)	*Maximum possible Score*	Colombi et al., (79)
		Context and setting. ***The study provides sufficient information regarding the critical features of the context or setting.***				
B	1.1	Study context/setting. yes=1, no=0	1	1	1	1
		Participants. ***The study provides sufficient information to identify the population of participants to which results may be generalized and to determine or confirm.***				
B	2.1	Participants demographics. yes=1, no=0	1	1	1	1
B	2.2	Participants disability/condition. yes=1, no=0	1	1	1	1
		Intervention agent. ***The study provides sufficient information regarding the critical features of the intervention agent.***				
B	3.1	Role of intervention agent. yes=1, no=0	1	1	1	1
B	3.2	Training of intervention agent. yes=1, no=0	1	1	1	1
		Description of practice. ***The study provides sufficient information regarding the critical features of the practice (intervention), such that the practice is clearly understood and can be reasonably replicated.***				
B	4.1	Intervention procedures. yes=1, no=0	1	1	1	1
B	4.2	Intervention materials (when relevant). yes=1, no=0	1	0	1	0
		Implementation fidelity. ***The practice is implemented with fidelity.***				
B	5.1	Fidelity (adherence). yes=1, no=0	1	1	1	1
B	5.2	Fidelity (dosage/exposure). yes=1, no=0	1	1	1	1
B	5.3	Fidelity (regularly/interventionist/setting/participant) (if 5.1 and 5.2 = no, then this item is NA). yes=1, no=0	1	0	1	1
		Internal validity. ***The independent variable is under the control of the experimenter. The study describes the services provided in control and comparison conditions and phases. The research design provides sufficient evidence that the independent variable causes a change in the dependent variable or variables. Participants stayed with the study, so attrition is not a significant threat to internal validity.***				
B	6.1	Independent variable. yes=1, no=0	1	1	1	1
B	6.2	Describe baseline/control conditions. yes=1, no=0	1	1	1	1
B	6.3	Baseline/Control conditions have no access to the intervention. yes=1, no=0	1	1	1	NA
G	6.4 Between-group	Group allocation. yes=1, no=0	1	1	NA	NA
S	6.5 Single-subject	At least, three different evaluation timepoints. yes=1, no=0	NA	NA	1	1
S	6.6 Single-subject	Three different baseline data points. yes=1, no=0	NA	NA	1	1
S	6.7 Single-subject	Controlling for threats to internal validity. yes=1, no=0	NA	NA	1	1
G	6.8 Between-group	Low overall attrition. yes=1, no=0	1	0	NA	NA
G	6.9 Between-group	Low differential attrition. yes=1, no=0	1	0	NA	NA

		Outcome measures/dependent variables. ***Outcome measures are applied appropriately to gauge the effect of the practice on study outcomes. Outcome measures demonstrate adequate psychometrics.***				
B	7.1	Outcomes are socially important. yes=1, no=0	1	1	1	1
B	7.2	Definition and description of measurement of dependent variables. yes=1, no=0	1	1	1	1
B	7.3	All dependent variables are reported in the results. yes=1, no=0	1	1	1	1
B	7.4	The frequency and timing of outcome measures are appropriate. yes=1, no=0	1	1	1	1
B	7.5	Adequate internal reliability of dependent variables. yes=1, no=0	1	0	1	0
G	7.6 Between-group	Adequate validity of dependent variables. yes=1, no=0	1	1	NA	NA
		Data Analysis. ***Data analysis is conducted appropriately. The study reports information on effect size.***				
G	8.1 Between-group	Appropriate statistical analysis. yes=1, no=0	1	1	NA	NA
S	8.2 Single-subject	Graph results. yes=1, no=0	NA	NA	1	1
G	8.3 Between-group	Effect sizes. yes=1, no=0	1	0	NA	NA
		Total	24	18	22	19
				**75%**		**79.2%**

Note. B = applies to both group comparison and single-subject research studies; G = indicator applies only to group comparison studies; S = indicator applies only to single-subject research studies.

NA: not applicable.
